# Características clínicas e inmunológicas de pacientes con déficit de anticuerpos específicos contra antígenos polisacáridos en un hospital pediátrico de Colombia

**DOI:** 10.7705/biomedica.7562

**Published:** 2024-12-23

**Authors:** Lina M. Castaño-Jaramillo, Alejandra Munévar, Andrea Carolina Marín, Milena Villamil, Sonia Restrepo, Natalia Vélez

**Affiliations:** 1 Servicio de Inmunología Clínica y Alergia Pediátrica, Fundación Hospital Pediátrico de La Misericordia - HOMI, Bogotá, D. C., Colombia Fundación Hospital Pediátrico de La Misericordia - HOMI Fundación Hospital Pediátrico de La Misericordia - HOMI Bogotá, D. C. Colombia; 2 Neumología Pediátrica, Universidad El Bosque, Bogotá, D. C., Colombia Universidad El Bosque Universidad El Bosque Bogotá, D. C. Colombia; 3 Pediatría, Universidad Militar Nueva Granada, Bogotá, D. C., Colombia Universidad Militar Nueva Granada Universidad Militar Nueva Granada Bogotá, D. C. Colombia; 4 Servicio de Neumología Pediátrica, Fundación Hospital Pediátrico de La Misericordia - HOMI, Bogotá, D.C., Colombia Fundación Hospital Pediátrico de La Misericordia - HOMI Fundación Hospital Pediátrico de La Misericordia - HOMI Bogotá, D.C. Colombia; 5 Departamento Pediatría, Universidad Nacional de Colombia, Bogotá, D.C., Colombia Universidad Nacional de Colombia Universidad Nacional de Colombia Bogotá, D.C. Colombia

**Keywords:** enfermedades de inmunodeficiencia primaria, síndromes de inmunodeficiencia, inmunoglobulinas, anticuerpos, neumonía, niño, Primary immunodeficiency diseases, immunologic deficiency syndromes, immunoglobulins, antibodies, pneumonia, child

## Abstract

**Introducción.:**

La deficiencia de anticuerpos específicos es un tipo de error innato de la inmunidad humoral, caracterizado por niveles normales de isotipos de inmunoglobulinas, infecciones recurrentes y reducción en la reacción vacunal ante antígenos polisacáridos.

**Objetivo.:**

Describir las características clínicas e inmunológicas de pacientes con déficit de anticuerpos específicos, atendidos en un hospital pediátrico de Bogotá entre mayo de 2021 y septiembre de 2023.

**Materiales y métodos.:**

Se revisaron las historias clínicas de 16 pacientes con déficit de anticuerpos específicos.

**Resultados.:**

La edad mediana al diagnóstico fue de seis años y medio, 9 pacientes eran de sexo masculino y 7 tenían antecedentes de prematuridad. Once tenían un estado nutricional normal y 7 tenían una talla adecuada. La infección recurrente más frecuente fue la neumonía en 12 pacientes y en más de la mitad, se acompañó de alguna complicación. El fenotipo más frecuente fue el moderado y 15 recibieron inmunoglobulina como tratamiento definitivo.

**Conclusión.:**

La deficiencia de anticuerpos específicos es una alteración funcional del sistema inmunológico frecuentemente subdiagnosticada. Se debe sospechar en pacientes con otitis media y neumonías recurrentes o complicadas por choque séptico, derrame pleural o neumonía necrosante.

## Abstract (en)

**Introduction.:**

Specific antibody deficiency is an innate error of humoral immunity characterized by normal levels of immunoglobulin isotypes, recurrent infections, and a reduced reaction to polysaccharide antigens in vaccines.

**Objective.:**

To describe the clinical and immunological characteristics of patients with specific antibody deficiency attending a pediatric hospital in Bogotá between May 2021 and September 2023.

**Materials and methods.:**

We reviewed the medical records of 16 patients with specific antibody deficiency.

**Results.:**

The median age at diagnosis was six and a half years. Nine were male, and 7 had a history of prematurity. Eleven patients had adequate nutritional status, and 7 had standard height. The most frequent recurrent infection was pneumonia, affecting 12 patients; more than half of them experienced some associated complications. The most common phenotype was moderate, and 15 of the individuals received immunoglobulin as definitive treatment.

**Conclusion.:**

Specific antibody deficiency is a frequently underdiagnosed functional alteration of the immune system. It should be suspected in patients experiencing recurrent otitis media and pneumonia or in cases complicated by septic shock, pleural effusion, or necrotizing pneumonia.

La alteración de la reacción de anticuerpos contra antígenos polisacáridos es un subtipo de error innato de la inmunidad, del grupo de las deficiencias de anticuerpos. Se conoce como deficiencia de anticuerpos específicos o deficiencia selectiva de anticuerpos ^1^ y se caracteriza por la incapacidad de generar una reacción efectiva de anticuerpos contra los antígenos purificados de polisacáridos capsulares de *Streptococcus pneumoniae*.

Usualmente, estos pacientes cuentan con niveles normales de inmunoglobulinas séricas G, A y M, y una adecuada reacción vacunal a los antígenos proteicos ^2^. La deficiencia de anticuerpos específicos se puede diagnosticar en pacientes mayores de dos años con antecedentes de infecciones recurrentes, como otitis, sinusitis o neumonías, a pesar de tener un esquema de vacunación completo con vacuna conjugada y polisacárida de neumococo. Por lo general, estos pacientes no logran generar anticuerpos protectores o memoria inmunológica a largo plazo ^3^, lo que altera la opsonización y fagocitosis de *S. pneumoniae*^4^. La incidencia de deficiencia de anticuerpos específicos varía entre el 6 y el 23 % en pacientes pediátricos con infecciones recurrentes, y es uno de los errores innatos de la inmunidad más frecuentes ^5^.

Se describen las características clínicas e inmunológicas de un grupo de pacientes con diagnóstico de inmunodeficiencia de anticuerpos específicos atendidos en un hospital pediátrico de referencia y alta complejidad en Bogotá.

## Materiales y métodos

### Diseño del estudio

Se realizó un estudio retrospectivo, observacional y descriptivo, en pacientes atendidos en la consulta ambulatoria de inmunología y neumología pediátrica de la Fundación Hospital Pediátrico de La Misericordia (HOMI) en Bogotá, Colombia. El estudio fue aprobado por el Comité de Ética del Hospital, en el acta 84 635-23.

Se incluyeron pacientes pediátricos con diagnóstico de deficiencia de anticuerpos específicos contra antígenos polisacáridos evaluados de mayo de 2021 a octubre de 2023.

Para las tablas de crecimiento y desarrollo, se utilizaron las directrices establecidas por la Organización Mundial de la Salud (OMS) y por el Ministerio de Salud y Protección Social de Colombia ^6^; asimismo, para las tablas de crecimiento para pacientes con síndrome de Down ^7^.

Se consideraron las siguientes definiciones de inmunodeficiencia de anticuerpos específicos ^1^^,^^2^:

- *Fenotipo grave*: pacientes con dos o menos serotipos protectores (≥1,3 μg/ml).
- *Fenotipo moderado*: menos del 70 % de serotipos protectores (≥ 1,3 μg/ml) en mayores de seis años y menos del 50 % (≥ 1,3 μg/ml) en pacientes entre 2 y 6 años.
- *Fenotipo leve*: que no tuvieron, al menos, una duplicación de los títulos prevacunales en mínimo el 70 % de los serotipos en mayores de 6 años, y al menos el 50 % de los serotipos en pacientes entre 2 y 6 años.
- *Fenotipo de pérdida de memoria*: pérdida de la respuesta inmunológica estándar inicial frente a polisacáridos en un período de 6 a 12 meses.

### Análisis estadístico

Se recolectaron datos relacionados con variables demográficas, clínicas e inmunológicas. Se hizo el análisis estadístico con el paquete de IBM™, SPSS Statistics, versión 29 ^8^, y se realizó la verificación mediante filtros. Se llevó a cabo un análisis univariado, en el cual las variables cualitativas se ordenaron en frecuencias absolutas, frecuencias relativas y porcentajes. Las variables cuantitativas se presentaron con medidas de tendencia central, como promedio y desviación estándar o medianas y rangos intercuartílicos, según la determinación de distribución de normalidad mediante el test de Shapiro-Wilk.

## Resultados

Se analizaron los datos de 16 pacientes con inmunodeficiencia de anticuerpos específicos, aislada, no asociada con otras alteraciones inmunológicas identificadas. La mediana de la edad al momento del diagnóstico fue de seis años y medio, con un ligero predominio del sexo masculino (9 pacientes). Siete de los pacientes tenían antecedente de prematuridad. El resto de las características de la cohorte estudiada se presentan en el cuadro 1.

**Cuadro 1:** Características de los pacientes con deficiencia de anticuerpos específicos (N = 16)

Variable		Mediana (RIQ)	n
		n	
Edad actual (años)		8 (3)	
Edad al diagnóstico (años)		6,5 (2)	
Sexo masculino			9
Estado nutricional			
	Eutróficos		11
	Riesgo de delgadez		2
	Delgadez		1
	Sobrepeso		1
	Obesidad		1
Talla (cm)			
	Talla adecuada		7
	Riesgo de talla baja		3
	Talla baja (Z score ≤ 2)		6
Infecciones			
	Otitis		5
	Sinusitis		5
	Neumonía		12
Atopia			
	Asma		14
	Rinitis alérgica		14
Autoinmunidad, colangitis esclerosante			1
Inmunoglobulinas séricas			
	IgA (mg/dl)	100 (81)	
	IgM (mg/dl)	128 (71)	
	IgG (mg/dl)	985 (392)	
	IgE (kU/L)	93 (372)	
Linfocitos B		830 (757)	
Fenotipo SAD			
	Leve		1
	Moderado		13
	Grave		1
	Pérdida de memoria		1

IQR: rango intercuartílico; SAD: inmunodeficiencia de anticuerpos específicos

En esta serie, se encontraron seis pacientes con síndromes genéticos asociados: dos con síndrome de Down, uno con síndrome de Rubinstein Taybi, uno con síndrome de Wiedemann Steiner, uno con discinesia ciliar primaria con deficiencia homocigota de DNAH11 y uno con miopatía por heterocigosis compuesta en el gen que codifica para la selenoproteína N (SELENON).

### Infecciones

La mayoría (n = 12) de los pacientes evaluados presentó neumonía: cinco, menos de tres episodios, tres, entre cuatro y nueve episodios, y cuatro, más de diez episodios. Ocho de los pacientes presentaron neumonía complicada: seis con choque séptico, uno con derrame pleural y uno con neumonía necrosante.

Se presentó otitis media aguda en 5 de 16 pacientes, uno presentó menos de tres episodios, dos, entre cuatro y nueve episodios, y dos, más de diez episodios. En ninguno de los pacientes se identificó alteración auditiva secundaria o antecedentes de mastoiditis. Cinco pacientes presentaron sinusitis, uno de ellos con absceso y empiema epidural secundario. Dos pacientes tenían asma grave, de difícil control, pero no cumplieron con los criterios de infecciones recurrentes. En una paciente se sospechó tuberculosis pulmonar por presentar tuberculina positiva y nódulos pulmonares en la tomografía de tórax, pero no se documentó el aislamiento de la micobacteria.

### Atopia y autoinmunidad

La mayoría de los pacientes cursó con rinitis y asma, De los 14 pacientes con asma, tres tenían antecedente de asma grave, potencialmente fatal. Seis pacientes presentaban alergia a ácaros del polvo doméstico y, dos, alergia concomitante a los gatos. En cuatro de los pacientes con asma, se evaluó la función pulmonar mediante espirometría u oscilometría: en tres, hubo un patrón obstructivo leve y, en el restante, un patrón restrictivo. De los nueve pacientes estudiados con tomografía de tórax, uno presentó bronquiectasias (11 %). De los 16 pacientes de esta serie, solo en uno se evidenció una enfermedad autoinmunitaria: colangitis esclerosante primaria.

### Abordaje inmunológico

Doce pacientes tenían niveles normales de las inmunoglobulinas G y A, mientras que 4 tenían, incluso, niveles elevados para su edad. La IgM se encontraba en valores normales en 14 pacientes y, elevada, en 2; 10 pacientes tenían niveles elevados de IgE.

Todos los pacientes tenían una alteración de la respuesta contra antígenos polisacáridos, con una mediana de serotipos protectores del 44 % (RIQ = 35). El fenotipo más frecuente fue el moderado en 13 de los pacientes; fue leve en uno, grave en uno y con pérdida de memoria en otro.

### Tratamiento

Cinco de los 16 pacientes recibieron azitromicina como tratamiento profiláctico de primera línea, pero presentaron infecciones repetitivas a pesar de recibirla, por lo cual, se les administró inmunoglobulinas. En un paciente, no se prescribió terapia para su inmunodeficiencia de anticuerpos específicos, ya que no presentó nuevos cuadros infecciosos después de hacerse el diagnóstico.

Quince pacientes recibieron suplemento con inmunoglobulinas (94 %), 12 por vía intravenosa y 3 por vía subcutánea. La mediana de la dosis de inmunoglobulina fue de 607 mg/kg (RIQ = 197). Con el suplemento de inmunoglobulinas, hubo mejoría subjetiva en los episodios infecciosos, con menor tiempo de hospitalización y menos necesidad de antibióticos.

## Discusión

La inmunodeficiencia de anticuerpos específicos es uno de los defectos inmunológicos más identificados en pacientes mayores de dos años y con infecciones sinopulmonares recurrentes ^9^. En este estudio, se presentan las características de 16 pacientes con esta enfermedad en un hospital pediátrico de alto nivel de complejidad en Colombia. En esta serie, se excluyeron quienes tenían un defecto cuantitativo de algún isotipo de inmunoglobulina o que tenían compromiso de linfocitos T o B. Lo anterior se hizo para ajustarse a los criterios diagnósticos europeos y norteamericanos ^10^.

El diagnóstico se hizo principalmente en la edad escolar, con una distribución similar en ambos sexos. La mayoría de los pacientes eran eutróficos, aunque en más de un tercio se encontró talla baja. Se encontraron síndromes genéticos asociados con la inmunodeficiencia en 6 de los pacientes; el más frecuente fue el síndrome de Down que frecuentemente puede asociarse con alteraciones de la inmunidad humoral ^11^.

También, se presentó un paciente con discinesia ciliar confirmada genéticamente por la presencia de una variante homocigota patógeca en el gen *DNAH11*. Cuando se le diagnosticó la inmunodeficiencia de anticuerpos específicos, se inició el suplemento de inmunoglobulinas, con lo cual se redujo significativamente el número de exacerbaciones pulmonares. En un estudio pequeño de Bélgica, se encontró que el 6,5 % de los pacientes con discinesia ciliar primaria tenía una afección inmunológica humoral asociada ^12^.

Las infecciones más frecuentes fueron las pulmonares, seguidas de las de las vías aéreas superiores. Se resalta que dos terceras partes de los pacientes con neumonía tuvieron complicaciones asociadas, como choque séptico, neumonía necrosante o derrame pleural.

En el contexto actual, Colombia cuenta con un esquema de vacunación conjugada para neumococo en el plan ampliado de inmunización, por lo cual la enfermedad invasiva por neumococo puede indicar la existencia subyacente de un error innato de la inmunidad. En un estudio prospectivo multicéntrico desarrollado en Australia, se encontró que el 12 % de los pacientes con infección neumocócica invasiva tenían alguna alteración inmunológica subyacente, predominantemente, defectos de anticuerpos; en el 1,4 % de los que se sometieron a evaluación inmunológica, se trataba de una deficiencia de anticuerpos específicos ^13^. En la presente serie, hubo una paciente con asma y tos crónica en quien se sospechó tuberculosis pulmonar, pero no se aisló la micobacteria. El déficit de anticuerpos específicos no es un error innato de la inmunidad que se asocie con predisposición a la infección con micobacterias ^14^.

En la presente serie, dos de los pacientes tenían títulos protectores (≥ 1,3 μg/ml) de inmunoglobulinas en más del 70 % de los serotipos. En uno de ellos, solo hubo duplicación de títulos en 13 de 23 serotipos, lo que corresponde a un fenotipo leve de inmunodeficiencia de anticuerpos específicos. En el otro paciente, hubo una reacción inicial satisfactoria, pero con pérdida de memoria en menos de un año, correspondiente al fenotipo de pérdida de memoria inmunológica. Por esta razón, lo ideal es obtener títulos de anticuerpos antes y después de la vacuna, para evaluar el incremento de los anticuerpos. Si el paciente continúa con infecciones a pesar de una primera seroconversión satisfactoria, se debe reevaluar la reacción inmunológica después de seis meses para descartar este fenotipo de pérdida de memoria inmunológica ^15^.

Además, en la presente serie se encontró una gran prevalencia de enfermedades atópicas asociadas: rinitis y asma en 14 pacientes, mucho más que lo reportado en otras series. En una cohorte con deficiencias humorales en Estados Unidos, se diagnosticó rinitis en el 11 % y asma en el 52 % de los casos ^16^. También, se observó que en 10 pacientes había elevación de los niveles de IgE, lo cual probablemente esté relacionado con la enfermedad alérgica asociada y con que la mayoría de los que contaban con pruebas de función pulmonar presentaron un patrón obstructivo.

Dos de los pacientes de esta serie tenían asma grave y, en ellos, se logró un mejor control pulmonar con menos exacerbaciones mediante la administración del suplemento de inmunoglobulina para la deficiencia de anticuerpos específicos, como se ha descrito anteriormente ^17^, se sospecha que estos pacientes podrían haber estado presentando infecciones subclínicas que perpetuaban la reacción inflamatoria pulmonar ^18^.

En 5 de los 16 pacientes, se inició un tratamiento profiláctico con azitromicina, pero fue necesario sustituirlo por suplemento de inmunoglobulinas debido a la persistencia de la infección. Las alternativas de tratamiento se deben contemplar según el fenotipo clínico del paciente y sus condiciones particulares ^15^.

Es importante recordar que los errores innatos de la inmunidad no son estacionarios y pueden evolucionar en el tiempo. Algunos autores han encontrado que, después de tener un diagnóstico inicial de inmunodeficiencia de anticuerpos específicos o deficiencia selectiva de IgA, algunos pacientes evolucionaron a un fenotipo de inmunodeficiencia común variable ^19^^,^^20^. En contraparte, también hay pacientes que, a pesar de documentarse una inmunodeficiencia de anticuerpos específicos después de vacunarse, no presentan nuevas infecciones; además, hay otros en quienes, después de un período de reposición con suplemento de inmunoglobulinas, se puede suspender el tratamiento sin recurrencia de complicaciones infecciosas ^15^.

En la presente serie, un paciente con inmunodeficiencia de anticuerpos específicos presentó infección crónica activa por virus de Epstein-Barr y, actualmente, está siendo reevaluado mediante estudios adicionales, mientras que, en otros pacientes, se ha podido suspender el suplemento de inmunoglobulinas sin que presenten nuevas infecciones hasta la actualidad.

Las limitaciones de este trabajo son que la población evaluada pertenece a un solo centro médico y que se trata de un estudio observacional retrospectivo de casos clínicos en un momento determinado en el tiempo, ya que el fenotipo clínico de los pacientes puede evolucionar, como ya se mencionó. También, se plantea la inquietud de que, con el advenimiento de nuevas vacunas conjugadas la seroconversión obtenida con la vacuna polisacárida contra neumococo podría no ser una adecuada herramienta diagnóstica en pacientes con esta enfermedad.

## Conclusión

La inmunodeficiencia de anticuerpos específicos es un defecto humoral que debe considerarse en pacientes con infecciones sinopulmonares graves o recurrentes. Estos pacientes pueden presentar niveles de inmunoglobulinas normales e incluso aumentados, pero con un defecto funcional en la respuesta contra los polisacáridos. Aquellos con enfermedad neumocócica invasiva o infecciones sinopulmonares recurrentes, deben ser evaluados para descartar un error innato subyacente de la inmunidad. La presencia de atopia o síndromes genéticos no excluye el diagnóstico de inmunodeficiencia de anticuerpos específicos.
